# Integrator Complex Subunit 3 Knockdown Has Minimal Effect on Lytic Herpes Simplex Virus Type-1 Infection in Fibroblast Cells

**DOI:** 10.17912/micropub.biology.001171

**Published:** 2024-05-15

**Authors:** Joseph R Heath, Daniel P Fromuth, Jill A Dembowski

**Affiliations:** 1 Department of Biological Sciences, Duquesne University, Pittsburgh, Pennsylvania, United States

## Abstract

Proteomic analysis of viral and cellular proteins that copurify with the herpes simplex virus type-1 (HSV-1) genome revealed that the cellular Integrator complex associates with viral DNA throughout infection. The Integrator complex plays a key role in the regulation of transcription of cellular coding and non-coding RNAs. We therefore predicted that it may regulate transcription of viral genes. Here, we demonstrate that knockdown of the Integrator complex subunit, Ints3, has minimal effect on HSV-1 infection. Despite reducing viral yield during low multiplicity infection, Ints3 knockdown had no effect on viral DNA replication, mRNA expression, or yield during high multiplicity infection.

**Figure 1. Integrator complex subunits colocalize with viral genomes, but Ints3 is not necessary for high multiplicity infection of fibroblast cells:**
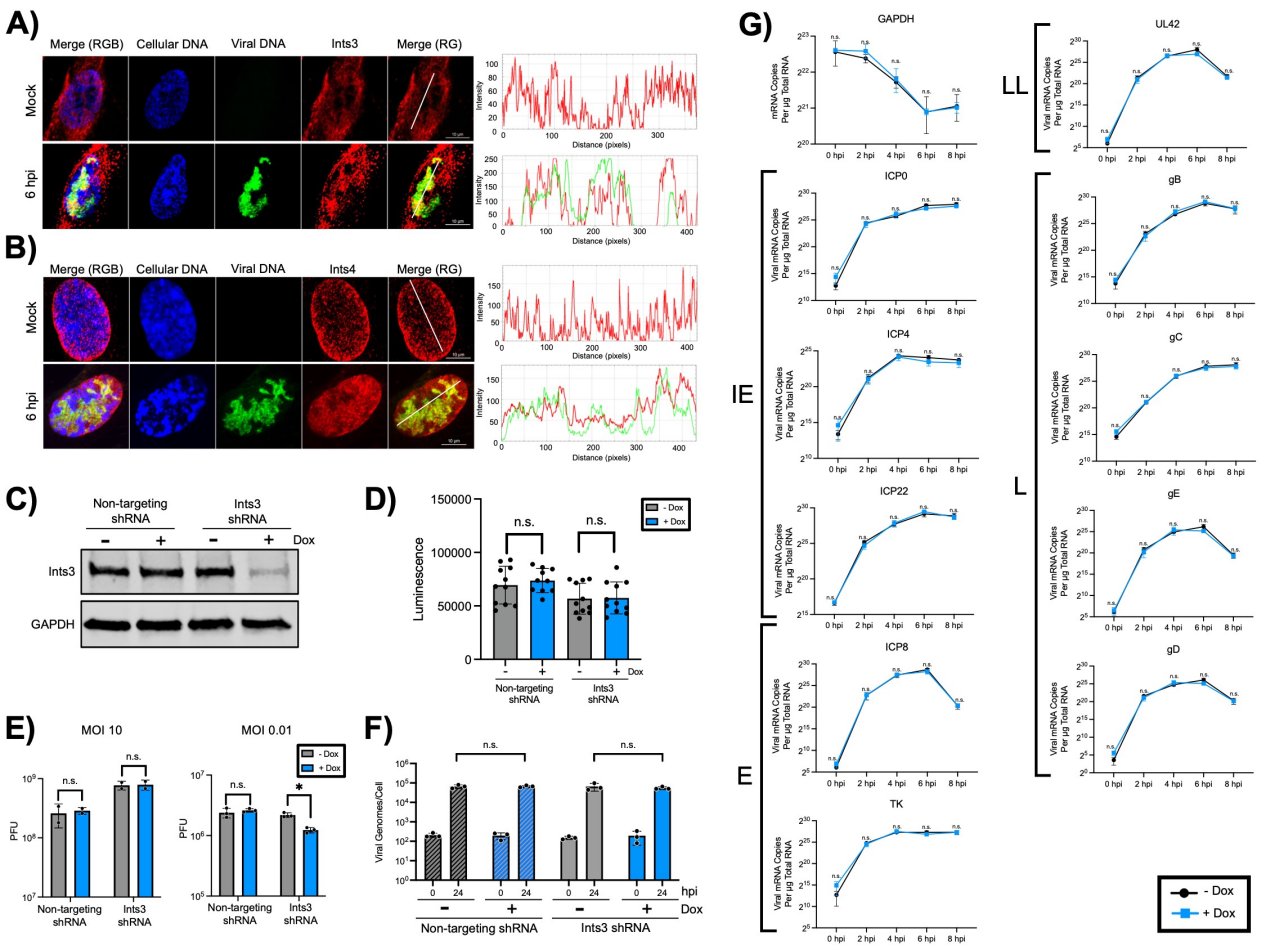
Confocal microscopy confirms that Ints3 (A) and Ints4 (B) colocalize with 5'-ethynyl-2'-deoxycytidine (EdC)-labeled HSV-1 genomes at 6 hours post infection (hpi). MRC-5 cells were infected at a multiplicity of infection (MOI) of 10 plaque forming units (PFU) per cell, replicating viral genomes were labeled with EdC from 4-6 hpi, and cells were fixed at 6 hpi. Viral genomes were labeled by click chemistry (green), cellular DNA by DAPI stain (blue), and Integrator subunits by indirect immunofluorescence (red). C) Western blotting confirms that Ints3 can be successfully knocked down in BJ-5ta fibroblast cells that express a doxycycline (dox) inducible Ints3-targeting short hairpin RNA (shRNA), with no knockdown occurring in a control cell line that expresses a dox-inducible non-targeting shRNA. D) Ints3 knockdown does not alter cell viability as confirmed by the CellTiter-Glo® Luminescent Cell Viability Assay. E) Ints3 knockdown reduces viral yield at an MOI of 0.01 PFU/cell, but not at an MOI of 10 PFU/cell. Ints3 and non-targeting shRNA cells were infected in the presence and absence of dox. At 24 hpi, virus was isolated and titered via plaque assay. F) Ints3 knockdown does not alter viral DNA replication as determined by quantitative PCR (qPCR) of the viral thymidine kinase (TK) and cellular glyceraldehyde 3-phosphate dehydrogenase (GAPDH) genes from total DNA isolated from cells infected at an MOI of 10 PFU/cell at 0 and 24 hpi. G) Ints3 knockdown does not affect the expression of select viral genes. The quantity of select viral immediate early (IE), early (E), leaky late (LL), and late (L) gene transcripts and cellular GAPDH were calculated by qPCR of reverse transcribed polyA RNA isolated from cells infected at an MOI of 10 PFU/cell at indicated times. All included statistical analyses were Student’s *t* -tests, where n.s. indicates a p value > 0.05 and * indicates a p value < 0.05.

## Description

HSV-1 is a ubiquitous double-stranded DNA virus. HSV-1 infection can result in a variety of disease manifestations including cold sores, ocular infection, or encephalitis (Alsweed et al., 2018; Bradshaw & Venkatesan, 2016; Kaye & Choudhary, 2006; Worrall, 2009) . During infection, HSV-1 undergoes a highly ordered temporal cascade of gene expression. IE genes are expressed upon entry of the viral DNA into the nucleus, followed by E genes and the onset of LL gene expression. Subsequently, DNA replication begins, resulting in the amplification of LL genes and the onset of L gene expression (Arvin, 2007; Dremel & DeLuca, 2019a; Harkness et al., 2014; Holland et al., 1980; Honess & Roizman, 1974, 1975; Jones & Roizman, 1979; Powell et al., 1975; Zhang & Wagner, 1987) . As is the case with many viruses, HSV-1 employs both viral and cellular factors to facilitate infection. Some of these factors have essential roles in infection, such as human RNA polymerase II (Pol II), which is required for transcription of viral genes (Alwine et al., 1974; Ben-Zeev & Becker, 1977) . Several additional host proteins that associate with the viral genome throughout infection have been identified, but do not have a defined role in the infection process (Dembowski & DeLuca, 2015, 2018; Dembowski et al., 2017; Kim et al., 2021; Reyes et al., 2017) . One group of proteins that falls into this category are members of the Integrator complex. Integrator complex members associate with viral genomes throughout infection and have been found to copurify with both infecting (Dembowski & DeLuca, 2018) and replicating (Dembowski et al., 2017) viral DNA. As host factors such as transcription factor IID (TFIID), TATA binding protein (TBP), and the Mediator complex help to facilitate HSV-1 transcription regulation (Dembowski & DeLuca, 2018; Dremel & DeLuca, 2019a, 2019b; Lester & DeLuca, 2011; Sampath & Deluca, 2008; Wagner & DeLuca, 2013) , it is possible that the Integrator complex also contributes to this process.

The Integrator complex is a metazoan-specific protein complex that consists of 15 subunits (Baillat et al., 2005; Offley et al., 2023) and plays many roles in transcription regulation (Welsh & Gardini, 2023) . Integrator carries out the endonucleolytic cleavage of the 3´ ends of several noncoding and protein-coding RNAs including small nuclear RNAs (snRNAs) (Baillat et al., 2005) , enhancer RNAs (eRNAs) (Lai et al., 2015) and mRNAs of replication-dependent histones (Skaar et al., 2015) . Integrator also contributes to the pause and release of Pol II through interactions with negative elongation factor (NELF), DRB sensitivity-inducing factor (DSIF), and transcription elongation factor b (pTEFb) (Fianu et al., 2021; Gardini et al., 2014; Kwak & Lis, 2013; Li et al., 2013; Stadelmayer et al., 2014) . In addition, the cleavage of short nascent transcripts by the Integrator complex results in the clearing of paused Pol II to facilitate transcription elongation (Beckedorff et al., 2020; Elrod et al., 2019a, 2019b; Tatomer et al., 2019) . Two Integrator subunits, Ints3 and Ints6, can act outside of the complex and contribute to the repair of double strand breaks (DSBs) in DNA (Jia et al., 2021; Li et al., 2021; Skaar et al., 2009) . The Integrator complex was found to regulate the production of microRNAs (miRNAs) and snRNAs coded for by the primate virus, Herpesvirus saimiri (HVS). Integrator cleaves the 3´ ends of HVS snRNAs, along with the 5´ end of adjacent pre-miRNAs immediately downstream from the viral snRNA genes (Cazalla et al., 2011) . The Integrator complex also acts through a transcription-independent mechanism to facilitate the 3´ end processing of HVS pre-miRNAs (Xie et al., 2015) . In a recent study, we found that inhibition of proliferating cell nuclear antigen (PCNA) with T2AA results in a decrease in Ints3 and Ints4 association with the HSV-1 genome at 6 hpi and a decrease in late viral gene expression (Packard et al., 2023) . Based on these observations, we hypothesized that the Integrator complex plays a role in the regulation of the transcription of HSV-1 genes. Ints3 is a suitable subunit to focus on in this context because of the dual contributions it makes to Integrator activity, both within and outside of the complex, as well as our lab’s recent findings relating to Ints3 association with the viral genome when PCNA is inhibited. Here, we discuss findings regarding the role of Ints3 in HSV-1 infection.

We first used confocal imaging of infected MRC-5 fibroblast cells to confirm Integrator complex colocalization with the HSV-1 genome. At 6 hours post infection (hpi), we observed Ints3 ( Figure 1A ) and Ints4 ( Figure 1B ) colocalization with EdC-labeled HSV-1 DNA, supporting previous results that the Integrator complex copurifies with HSV-1 genomes (Dembowski & DeLuca, 2018; Dembowski et al., 2017) . To test the hypothesis that Ints3 is important for HSV-1 transcription regulation, we prepared cell lines that can be induced by doxycycline (dox) to express an Ints3 shRNA or a non-targeting shRNA control. We confirmed that the dox induction of Ints3 shRNA expression, but not the non-targeting control, resulted in an approximately 3-fold reduction in Ints3 protein levels via western blotting ( Figure 1C ). This knockdown is highly reproducible and consistent among all experiments. To ensure that Ints3 knockdown did not result in cell death, we conducted a CellTiter-Glo® Luminescent Cell Viability Assay, which measures the ATP levels of metabolically active cells, with more viable cells resulting in a higher luminescence signal. There was no difference in cell viability between the induced and uninduced conditions for both Ints3 shRNA cells and a non-targeting shRNA control ( Figure 1D ), validating this system for further experiments.

To determine the effects of Ints3 knockdown on HSV-1 infection, we determined the yield of progeny viruses produced in non-targeting and Ints3 shRNA cells in the presence and absence of dox. To study multistep growth and synchronized infection we infected at a low or high MOI, respectively. Virus was harvested from infected cells at 24 hpi and titered in Vero cells by plaque assay. At an MOI of 0.01 PFU/cell, there was a statistically significant, though modest, 1.77 fold decrease in the amount of virus produced. However, at an MOI of 10 PFU/cell, no change in yield was observed ( Figure 1E ). Following these observations, we next determined if Ints3 knockdown affects viral transcription and DNA replication. To study DNA replication, we infected both induced and uninduced Ints3 and non-targeting shRNA cells and harvested total DNA at 0 and 24 hpi. We then used qPCR to determine the number of viral and cellular genomes in order to calculate the number of viral genomes per cell. Ints3 knockdown had no effect on viral DNA replication at a high MOI ( Figure 1F ). Finally, to examine the effect of Ints3 knockdown on transcription, we isolated total RNA from infected induced or uninduced shRNA cells. Using qRT-PCR, we examined the expression of representative transcripts from each viral gene class along with GAPDH as a cellular transcription control. Ints3 knockdown had no effect on the expression of any genes examined regardless of the temporal gene class ( Figure 1G ). The absence of a transcription defect here aligns with data in Figures 1E and 1F, as infection at an MOI of 10 did not affect either viral yield or DNA replication. As these samples were infected at a high MOI, it is possible that small differences could be amplified by examining lower amounts of infecting virus. It is also conceivable that a sequencing-based approach could provide a more global understanding of how this knockdown alters viral transcription, including both coding and non-coding transcripts.

Taken together, these results indicate that Ints3 plays a minimal role in the HSV-1 infection cycle. While we observed that knockdown results in a decrease in viral yield, these results were at a low MOI. This suggests that Ints3 may serve a redundant or nonessential function with respect to infection. Both gene expression and DNA replication data support this, as knockdown did not result in a defect in either process. In addition, these data suggest that Ints3 does not play a major role in the DNA damage response during infection to either promote or inhibit infection under the experimental conditions tested in this study. It is possible, however, that the residual Ints3 remaining after knockdown could have still allowed the Integrator complex to function enough to support viral transcription and DNA replication. Despite these findings, it is still possible that the Integrator complex plays a yet to be identified role in HSV-1 infection because it is key to the regulation of transcription by Pol II and copurifies and colocalizes with HSV-1 DNA ( Figure 1A, B ). Ints3 is not a member of the catalytic core of the Integrator complex, which includes Ints4, Ints9, and Ints11. It would therefore be interesting to investigate the functions of these complex members in HSV-1 transcription regulation in the future.

## Methods

**Immunofluorescence Imaging**

MRC-5 cells were plated at a density of 1.67x10 ^5^ cells/well in a 12-well dish containing glass coverslips in Dulbecco’s modified Eagle medium (DMEM, Gibco) supplemented with 10% fetal bovine serum (FBS, Gibco). Cells were infected at a MOI of 10 PFU/cell and incubated at 37°C for 4 hours, at which time medium was replaced with medium supplemented with 25 µM EdC. Coverslips were incubated for another 2 hours, then fixed with 4% paraformaldehyde for 15 min at room temperature. Coverslips were stored at 4°C overnight in phosphate buffered saline (PBS) containing 3% bovine serum albumin (BSA). Cells were permeabilized with 0.5% Triton X-100 in PBS for 20 minutes and then blocked with PBS containing 3% BSA for 30 minutes, followed by the addition of the click reaction for 30 minutes (Dembowski & DeLuca, 2017) . Cells were then incubated in DAPI stain for 30 minutes, followed by another 30 minute incubation with a 1:200 dilution of rabbit α-Ints3 (Proteintech 16620-1-AP) or a 1:500 dilution of rabbit α-Ints4 (Bethyl A301-296A) antibodies. The cells were washed in PBS containing 1% BSA followed by three subsequent washes with PBS. Alexa-Fluor 594-conjugated α-rabbit IgG (Invitrogen A-11012) was added to the cells at a 1:200 dilution for 30 min, followed by another series of washes with PBS. The coverslips were mounted onto a slide using Shandon™ Immu-Mount™ and imaged with a Nikon Eclipse Ti2 Inverted Confocal Microscope. Traces were generated using the RGB profiler plugin in ImageJ.

**Doxycycline Inducible shRNA Cell Line Generation**

Doxycycline Inducible shRNA cells were generated in BJ-5ta cells as indicated (Wee et al., 2008; Wiederschain et al., 2009) . BJ-5ta cells, which are immortalized, were used for these experiments in order to create stable cell lines. Specific sequences used for generation of cell lines are listed in Table 1.

**Doxycycline Induction of shRNA Cells**

Ints3 shRNA or non-targeting shRNA cell lines were plated in DMEM supplemented with 10% FBS, 0.5 µg/mL puromycin (Fisher Scientific), and 0.01mg/mL hygromycin B (Life Technologies). The following day, medium was replaced with DMEM supplemented with 10% FBS and 1µg/mL doxycycline (Fisher Scientific). At 24 and 48 hours posts-induction, 1 µg dox was added. At 72 hours post-induction, medium was replaced with fresh DMEM supplemented with 10% FBS containing 1 µg/mL doxycycline. As a control, cell lines were also grown in the absence of dox and were subject to a medium change at 72 hours post-induction. Experiments were carried out at 96 hours post-induction.

**Western Blotting**

Ints3 or non-targeting shRNA cell lines were plated at a density of 2.5x10 ^5^ cells/well in a 12-well dish and induced as described above. Cells were harvested in 2x Laemmli sample buffer (100 mM Tris pH 6.8, 20% glycerol, 4% sodium dodecyl sulfate (SDS), 0.02% bromophenol blue) and subject to SDS-polyacrylamide gel electrophoresis and western blotting. The following primary antibodies were used for western blotting: rabbit α-Ints3 (Proteintech 16620-1-AP, 1:500 dilution) and mouse α-GAPDH (Invitrogen AM4300, 1:5,000 dilution). Blots were imaged using a LI-COR Odyssey FC Imager.

**CellTiter-Glo® Luminescent Cell Viability Assay**

Ints3 or non-targeting shRNA cells were plated at a density of 1.65x10 ^4^ cells/well in a 96-well plate in DMEM supplemented with 10% FBS, 0.5 µg/mL puromycin, and 0.01mg/mL hygromycin B. Dox induction was carried out as described and at 96 hours post-induction, cells were subject to the CellTiter-Glo® Luminescent Cell Viability Assay (Promega) as indicated in the manufacturer’s protocol. A SpectraMax iD3 plate reader was used to measure luminescence.

**Viral Yield**

Ints3 or non-targeting shRNA cells were plated at a density of 2.5x10 ^5^ cells/well in a 12-well dish and induced or left uninduced as described above. At 96 hours post-induction, cells were infected with HSV-1 strain KOS at an MOI of 10 or 0.01 PFU/cell. Inoculum was prepared in tris buffered saline (TBS), which also included 1 µg/mL dox for the induced groups. Viruses were allowed to adsorb to the cells for 1 hour, then inoculum was aspirated. Cells were washed with warm TBS, then DMEM containing 10% FBS was added. Induced groups received medium supplemented with 1 µg/mL dox for the infection period (24 hours, 37ºC). After infection, cells and growth medium were collected and subject to 3 freeze-thaws and sonicated to release cell associated virus. Viral yield for each condition was determined via plaque assay in Vero cells.

**Viral Replication Assay**

Ints3 or non-targeting shRNA cells were plated at a density of 5x10 ^5^ cells/well in a 6-well dish and induced or left uninduced as described above. At 96 hours post induction, cells were infected at an MOI of 10 PFU/cell, following the same method described above. At 0 and 24 hpi, total DNA was harvested in DNA extraction buffer (0.5% SDS, 400 µg/mL proteinase K, 100 mM NaCl). qPCR was then used to measure the number of viral and cellular genomes. For viral genome quantification, primers that recognize the TK gene were used and genome number was quantified based on a standard curve generated from known amounts of viral DNA. For cellular genome quantification, primers specific for the GAPDH gene were used and quantities were determined relative to a standard curve generated from known amounts of human DNA. Primer sequences are listed in Table 2 (Garvey et al., 2014) .

**Gene Expression**

Ints3 shRNA cells were plated at a density of 2.5x10 ^5^ cells/well in a 12-well dish and induced or left uninduced as described above. Ninety-six hours post induction, cells were infected at an MOI of 10 PFU/cell as described above. Total RNA was isolated using Trizol reagent (Invitrogen) at 0, 2, 4, 6, and 8 hpi. Total RNA was isolated as indicated in the Trizol manufacturer’s protocol. Total RNA was reverse transcribed using Oligo(dT) Primers (Thermo Fisher Scientific). cDNA was then used for qPCR. GAPDH, ICP0, ICP4, ICP22, ICP8, TK, UL42, glycoprotein B (gB), gC, gE, and gD were examined using the primers listed in Table 2. The number of mRNA copies for each transcript was determined relative to standard curves generated from known quantities of purified HSV-1 or human DNA. Primer sequences listed in Table 2 are from Garvey et al, 2014 (Garvey et al., 2014) .

Table 1: Oligos used to clone Ints3-targeting and non-targeting shRNA sequences into the pLKOTet-On vector

**Table:** 

**shRNA Cell Line**	**Strand**	**Sequence (5´-3´)**
Ints3 shRNA	Top	CCGGAGTCGTGATGGCATGAATATTCTCGAGAATATTCATGCCATCACGACTTTTTT
	Bottom	AATTAAAAAAGTCGTGATGGCATGAATATTCTCGAGAATATTCATGCCATCACGACT
Non-targeting shRNA	Top	CCGGCCTAAGGTTAAGTCGCCCTCGCTCGAGCGAGGGCGACTTAACCTTAGGTTTTT
	Bottom	AATTAAAAACCTAAGGTTAAGTCGCCCTCGCTCGAGCGAGGGCGACTTAACCTTAGG

Table 2: Primer sequences used for qPCR

**Table:** 

**Gene**	**Forward Primer (5´-3´)**	**Reverse Primer (5´-3´)**
ICP0	GTCGCCTTACGTGAACAAGAC	GTCGCCATGTTTCCCGTCTG
ICP4	CGGTGATGAAGGAGCTGCTGTTGC	CTGATCACGCGGCTGCTGTACA
ICP22	ATGCAATGCTACGGCGCTCGGT	ACAGCTGATTGATACACTGGCGC
ICP8	CATCAGCTGCTCCACCTCGCG	GCAGTACGTGGACCAGGCGGT
TK	TCGATGTGTCTGTCCTCCG	ATCCCATCGCCGCCCTC
UL42	ACGTCCGACGGCGAGG	CAGGCGCAACTGAACGTC
gB	TACTGCGGCTGGCCCACCTTG	GCTCTCGCGCGTGGACCTG
gC	GAGGAGGTCCTGACGAACATCACC	CCGGTGACAGAATACAACGGAGG
gE	CGAGGACGTTTCGTTGCTTCC	GAGACCCACGACGGGTGTAA
gD	CTATGACAGCTTCAGCGCCGTCAG	CGTCCAGTCGTTTATCTTCACGAGC
GAPDH	CAGAACATCATCCCTGCCTCTACT	GCCAGTGAGCTTCCCGTTCA
